# Health care seeking delay among pulmonary tuberculosis patients in North West zone of Tigrai region, North Ethiopia

**DOI:** 10.1186/s12879-019-3893-7

**Published:** 2019-04-05

**Authors:** Haileselasie Berhane Alema, Sisay Asgedom Hailemariam, Kebede Haile Misgina, Meresa Gebremedhin Weldu, Yosef Sibhatu Gebregergis, Genet Kidane Mekonen, Kahsay Amare Gebremedhin

**Affiliations:** 1 Department of Public Health, College of Health Science, Aksum University, P.O.Box: 298, Axum, Ethiopia; 2 Asgede Tsimbla Woreda Health Office, Tigrai, Ethiopia; 3 Aksum University Comprehensive Specialized Hospital, Axum, Ethiopia; 4 College of medicine and health science, Adigrat University, Adigrat, Ethiopia

**Keywords:** Delay, Health care seeking, Pulmonary tuberculosis, Tigrai

## Abstract

**Background:**

Tuberculosis (TB) is a major public health problem and it is among the top 10 causes of death worldwide. One of the challenges against attaining an effective TB control program is delay in seeking health care to diagnosis and treatment of TB patients. The aim of this study was to assess health care-seeking delay among pulmonary TB patients.

**Methods:**

An institutional based cross-sectional study was conducted among new pulmonary tuberculosis (PTB) patients > 15 years of age who were enrolled in the intensive phase TB treatment from November 1, 2015 – January 30, 2016. Data were collected by an interviewer administered technique using a structured questionnaire. Health care seeking delay was categorized by using a median cutoff point of > 30 days as a prolonged health care seeking delay. Logistic regression analyses were employed to determine factors independently associated with the delays in health care seeking.

**Results:**

A total of 422 PTB patients were included in this study. The median age of respondents was 37 years (interquartile range (IQR) =35–44). The median time of health care seeking delay was 30 days (IQR) = 21-60). Respondents occupation, knowledge about pulmonary tuberculosis, health facility visited first, seeking treatment from traditional or religious healers before visiting health facilities, reason for not seeking treatment early from health facilities, and reason for first consultation were found to be significantly associated with health care seeking delay.

**Conclusion:**

The study showed the magnitude of health care seeking delay among pulmonary tuberculosis patient was very long and the factors associated with health care seeking delay were: occupation, knowledge status, health facility visited first and seeking treatment from religious or traditional healer before health facilities. To overcome delay of health care seeking among tuberculosis patients, efforts should required availing tuberculosis diagnostic and treatment services at the primary health care level.

## Background

Tuberculosis (TB) is a major public health issue worldwide. According to the WHO report 2018, globally TB is one of the top 10 causes of death and the leading cause mortality from a single infectious agent specifically in developing countries with 10 million new TB cases and 1.3 million deaths worldwide [1].

Ethiopia is among the 30 high TB and multidrug resistant(MDR-TB) burden countries, that accounting for 80% of all estimated TB cases worldwide, with an annual estimated TB incidence of 172/1000 populations and a death rate of 25 per 1000 population for 2017 [2] .

One of the challenges against attaining an effective TB control program is delay in seeking health care to diagnosis and treat of TB patients. It is predicted that an infected person who delays diagnosis and remains untreated, can transmit the disease to between 10 and 15 people per year [3].

Delay in TB diagnosis and treatment plays a major role in increasing the size of the infectious pool of TB which enhances transmission in the community. Care seeking delay worsens the disease by increasing the risk of drug resistance, treatment failure, mortality and transmission of drug resistant TB strains in the community [4–6]. Therefore, to reduce the burden of tuberculosis there should be more effective detection and cure rate, which can be achieved by early diagnosis and treatment of pulmonary tuberculosis. However, due to poor health seeking behavior, there is a long delay in diagnosis and treatment among pulmonary tuberculosis patients in low and middle income countries (LMICs) [4]; which is an average total delay, patient delay, and health system delay of 31.7 days, 28.4 days and 67.8 days respectively.

According to recent studies in Ethiopia, the median health care seeking delay length may ranges from 20 to 63 days [7–11]. Another study conducted in the Tigrai region, where the present study carried out, showed that the median health care seeking delay reported is 60 days [12]. This implies that there were no consistent data which specify the magnitude of health care seeking delay behaviors in the Tigrai region.

In developing countries like Ethiopia, where the economic status of the population is poor, literacy rate is low and basic health service access is scarce; studying determinants of health care seeking delay among PTB patients is critical to prevent severe and complications of the disease. Meanwhile, little work has been done on health care seeking delay among PTB patients that help program coordinators to develop strategies and guidelines likely to improve care seeking behaviors. Therefore, the aim of this study was to investigate the status of health care seeking delay and its predictors among pulmonary tuberculosis patients in the North-West zone of Tigrai region, North Ethiopia.

## Methods

### Study design, setting and population

An institutional based cross-sectional study was conducted among new pulmonary tuberculosis patients age > 15 years who enrolled on intensive phase from November 1, 2015– January 30, 2016 in selected public health institutions of North-West zone of Tigrai region, North Ethiopia. This zone is one of the seven zones found in the regional state and located 1087 km north direction of the capital Addis Ababa and 300 Km from capital of the regional state Mekelle. It has a total population of 889,756 and owns four governmental hospitals and 39 health centers, which provide directly observed treatment (DOT) services for pulmonary tuberculosis patients. Those who were unable to communicate, seriously ill as well as patients age below 15 years were excluded from the study.

### Sample size and sampling procedure

The sample size was determined using the single population proportion formula by assuming that: prevalence (p) of delay in health care seeking 50%, confidence level of 95%, margin of error of 5%, and non-response rate of 10%. As a result, the maximum sample size was calculated to be 422. Two hospitals and 19 health centers were selected randomly. Considering their average monthly patient flow received from the district health offices, the total sample size was proportionately allocated to each of the randomly selected public health facilities. Finally, respondents were consecutively enrolled until the required number is fulfilled in each health facility.

### Measurement

Data were collected by interviewer administered technique using a structured questionnaire adapted from other published sources [7–18]. In addition, medical records were reviewed for pulmonary tuberculosis (PTB) diagnostic information, such as date of first consultation, date of diagnosis, type of PTB, type of diagnostic investigation used to diagnose PTB, and date of treatment initiation.

Perceived TB stigma was assessed using 8 items rated on a three-point Likert scale as (1) disagree, (2) neutral, and (3) agree. The eight items adapted from other published sources [7, 15–17], and used to assess perceived PTB stigma include: (1) I feel ashamed for having PTB; (2) People have to hide PTB diagnosis from people; (3) PTB affects relation with people; (4) PTB affects the relationship with other family members; (5) I prefer to live isolated since I have diagnosed to have TB; (6) PTB affects work performance which can results in termination of employment; (7) PTB affects marital relations; and (8) People with PTB cannot cook for the family. The perceived stigma score was computed using the above 8 items whose theoretical value ranges from 8 to 24. Finally, respondents who scored above the median perceived stigma score of the sampled population were labeled as having high perceived stigma whereas respondents with median perceived stigma score of less than or equals to the median score were labeled as having low perceived stigma.

### Data quality management

The data were collected by eight diploma nurses and the data collection was entirely supervised by two senior BSc nurses. Furthermore, the data collection process was closely monitored by the principal investigators. Both data collectors and supervisors were trained for 2 days on the objectives of the study, sampling technique, ethical consideration, data collection tool and techniques of collecting data to maintain integrity of data collection throughout the study. The collected data were checked daily by the supervisors and feedback was provided to data collectors when necessary.

### Operational definition

**Health care seeking delay:** is the time from symptom onset to the first consultation. Symptom onset referred to the time at which the first symptom such as persistent cough, fever, weight loss and night sweats of the illness for which a patient seeking care began. Health care seeking delay was categorized by using a median cutoff point of 30 days. That is, > 30 days was taken as prolonged patient delay.

### Data processing and analysis

Data were entered in Epi-info version 7 and exported to the Statistical Package for Social Sciences (SPSS) version 21 for analysis. Descriptive statistics such as, frequency distribution, median, and interquartile range (IQR) were computed. The outcome variable was dichotomized and coded as “1” for unacceptable or longer delay and “0” for shorter delay. Bivariate analysis was used to identify the unadjusted effect of each factors on outcome variable of the study. The independent variables with a *p* < 0.05 in the bivariate analysis were fitted into a multivariate logistic regression model to identify their independent effect on outcome variable. Odds ratio with 95% confidence interval were calculated both to assess and measure the strength of association between the explanatory and outcome variables. Statistical significance was taken as *p* < 0.05.

## Results

### Socio-demographic characteristics

A total of 422 new PTB patients were included in this study. The median age of respondents was 37 years (IQR = 35–44). More than half 255 (60.4%) were males and nearly half 216 (51.2%) of the total respondents were rural residents. From the respondents, 389 (92.2%) were Orthodox by religion, 266 (63.0%) were married and 181 (42.9%) were farmers. Nearly half 212 (50.2%) had no formal education. More than three fourths (77.7%) of the patients live in less than an hour walking distance from the nearest health facility, where TB care is available (Table 1).

**Table 1:** Socio demographic characteristics of pulmonary tuberculosis patients in public health institutions, North-West zone of Tigrai region, North Ethiopia, November 2015 to January 2016 (*n* = 422)

Characteristics	Frequency (percent)
Age	
15–24	87 (20.6)
25–34	97 (23.0)
35–44	112 (26.5)
45–54	64 (15.2)
≥ 55	62 (14.7)
Sex	
Male	255 (60.4)
Female	167 (39.6)
Residence	
Urban	206 (48.8)
Rural	216 (51.2)
Marital status	
Married	266 (63.0)
Single	85 (20.1)
Widowed	26 (6.2)
Divorced	45 (10.7)
Religion	
Orthodox	389 (92.2)
Muslim	29 (6.9)
Protestant	2(.5)
Catholic	2(.5)
Occupation	
Farmer	181 (42.9)
Daily labor	56 (13.3)
Merchant	55 (13.0)
House wife	54 (12.8)
Civil servant	40 (9.5)
Student	36 (8.5)
Education	
Unable to read and write	157 (37.2)
Only read and write	55 (13.0)
Primary	91 (21.6)
Secondary and above	119 (28.2)
Family size	
< 5	255 (60.4)
≥ 5	167 (39.6)
Monthly family income	
< 500 ETB	90 (21.3)
500–999 ETB	152 (36.0)
1000–1499 ETB	68 (16.1)
≥ 1500 ETB	112 (26.5)
Accessibility of TB center (health center or hospital)	
≤ 1 h	328 (77.7)
> 1 h	94 (22.3)

### Knowledge and sources of information on pulmonary tuberculosis

Of the total respondents, only 177 (40.0%) knew correctly the cause of tuberculosis. Concerning the signs and symptoms of tuberculosis, the most commonly known sign and symptoms by the respondents were coughing for more than 2 weeks 338 (80.1%), sputum with blood 216 (51.2%), night sweating 208 (49.3%), fever 202 (47.9%) and loss of appetite 173 (41%). The vast majority 412 (97.6%) of the respondents knew that PTB is a transmissible disease. Pertaining to means of transmission, 354 (83.9%) respondents described PTB transmits through respiratory air droplets resulting from coughing/breathing /sneezing/ by the PTB patients. Likewise, nearly two thirds 254 (60.2%) knew that PTB is transmitted from PTB patient to healthy people by sharing drinking cups and 177 (42%) mentioned PTB can transmit by living with PTB patients in a crowded house.

Almost all of the respondents 421 (99.8%) believed that PTB is curable disease. Similarly, 409 (97%) of study participants stated that modern medicine is an effective treatment for PTB. Surprisingly, 60 (14.2%) believed that PTB can be treated with traditional healer or holy water.

### Perceived stigma

Respondents were asked to respond on eight stigmas related statements. Accordingly, nearly one in ten agreed to most of the statements; I feel ashamed for having PTB, people have to hide PTB diagnosis from people, PTB affects marital relations and people with PTB cannot cook for the family. Nearly half (51.9%) agreed that PTB affects the relation with people. Nearly two thirds agreed to the rest of the stigma related statements; PTB affects the relationship with other family members, I prefer to live isolated since I have been diagnosed to have PTB and PTB affect work performance which can result in termination of employment. Generally, a total of 252 (59.7%) of the total respondents had high perceived stigma related to PTB (Table 2).

**Table 2:** Perceived stigma related to pulmonary tuberculosis among pulmonary tuberculosis patients in public health institutions, North-West zone of Tigrai region, North Ethiopia, November 2015 to January 2016 (n = 422)

Characteristics	Disagree n (%)	Neutral n (%)	Agree n (%)
I feel ashamed for having PTB	219 (51.9)	39 (9.2)	164 (38.9)
People have to hide PTB diagnosis from people	189 (44.9)	43 (10.1)	190 (45.0)
PTB affects relation with people	152 (36.0)	51 (12.1)	219 (51.9)
PTB affects the relationship with other family members	117 (27.7)	32 (7.6)	273 (64.7)
I prefer to live isolated since I have diagnosed to have PTB	107 (25.4)	41 (9.7)	274 (64.9)
PTB affects work performance which can results in termination of employment	97 (23.0)	53 (12.5)	272 (64.5)
PTB affects marital relations	136 (32.2)	110 (26.1)	176 (41.7)
People with PTB cannot cook for the family	195 (46.2)	34 (8.1)	193 (45.7)
Composite score of perceived stigma related to PTB			
High	252 (59.7)		
Low	170 (40.3)		

### Health care seeking behavior

The median health care seeking delay was 30 days (IQR = 21-60). Of the total respondents, 328 (77.7%) were sought health care after 2 weeks. Likewise, 132 (31.3%) were delayed for more than 30 days before seeking health care. The longest delay was 360 days. Of the total 422 patients included in the study, only 200 (47.4%) sought health care from health facilities for the first time, whereas the rest tried other treatment modalities before their first consultation to health facilities. Thinking that their illness was not serious and trying other treatment modalities were the most common reasons for delayed consultation. Public health center or hospital was the health facility visited first by 340 (80.6%) of the respondents. Being seriously ill 349 (82.7%) and suspecting PTB 122 (28.9%) was the most commonly cited reasons for first consultation. The majority of PTB diagnoses were made in public hospitals 260 (61.6%) and health center 128 (30.3%). More than half 228 (54%) were diagnosed by sputum microscopic examination.

### Factors associated with health care seeking delay

Multivariate logistic regression was fitted to determine factors independently associated with health care seeking delay among pulmonary TB patients Table 3. Accordingly, being farmer [AOR = 3.38 (95% CI: 1.35, 8.49)], limited knowledge about pulmonary tuberculosis [AOR = 1.49 (95% CI: 1.14,2.26)], visiting public health center first [AOR = 0.42 (95% CI: 0.25, 0.70)], seeking treatment from traditional or religious healers before visiting health facilities [AOR = 1.71 (95% CI:1.07, 2.73)], reason for not seeking treatment early from health facilities for financial problem [AOR = 2.26 (95% CI: 1.26, 4.06)], and being severely ill with the diseases [AOR = 2.09 (95% CI: 1.10, 3.98)] were independent risk factors of health care seeking delay to diagnosis and treatment of pulmonary tuberculosis.

**Table 3:** Predictors of delay in seeking health care among pulmonary tuberculosis patients in public health institutions, North-West zone of Tigrai region, North Ethiopia, November 2015 to January 2016 (*n* = 422)

Variables	Health care seeking delay > 30 days)		COR (95% C.I)	AOR (95% C.I)
	Yes, n (%)	No, n (%)		
Age				
15–24	25 (18.9)	62 (21.4)	1	
25–34	21 (15.9)	76 (26.2)	0.69 (0.35,1.34)	
35–44	39 (29.5)	73 (25.2)	1.33 (0.72,2.43)	
45–54	23 (17.4)	41 (14.1)	1.39 (0.70,2.77)	
≥ 55	24 (18.2)	38 (13.1)	1.57 (0.79,3.12)	
Occupation				
Farmer	72 (54.5)	109 (37.6)	2.74 (1.14,6.58)	3.38 (1.35, 8.49)
House wife	12 (9.1)	42 (14.5)	1.18 (0.42,3.37)	1.37 (0.46, 4.08)
Merchant	16 (12.1)	39 (13.4)	1.70 (0.62,4.67)	2.03 (0.70, 5.95)
Civil servant	9 (6.8)	31 (10.7)	1.20 (0.40,3.65)	1.53 (0.48, 4.88)
Daily labourer	16 (12.1)	40 (13.8)	1.66 (0.60,4.54)	1.86 (0.65, 5.34)
Student	7 (5.3)	29 (10.0)	1	1
Knowledge				
Not knowledgeable	80 (60.6)	144 (49.7)	1.56 (1.33,1.84)	1.49 (1.142,2.262)
Knowledgeable	52 (39.4)	146 (50.3)	1	1
Perceived stigma				
No	46 (34.8)	139 (47.9)	1	
Yes	86 (65.2)	151 (52.1)	1.72 (1.12,2.63)	
Health facility visited first				
Private pharmacy/drug store	8 (6.1)	11 (3.8)	1.29 (0.49,3.40)	0.83 (0.29, 2.37)
Private clinic	24 (18.2)	31 (10.7)	1.38 (0.74,2.56)	1.13 (0.58, 2.21)
Public health post	1 (0.8)	7 (2.4)	0.25 (0.03,2.12)	0.17 (0.02, 1.47)
Public health center	40 (30.3)	136 (46.9)	0.52 (0.33,0.84)	0.42 (0.25, 0.70)
Public hospital	59 (44.7)	105 (36.2)	1	1
Sought treatment from traditional or religious healers before visiting health facilities				
Yes	83 (62.9)	139 (47.9)	1.84 (1.21,2.81)	1.71 (1.07, 2.73)
No	49 (37.1)	151 (52.1)	1	1
Reason for not seeking treatment early from health facilities				
Thinking the disease is easy				
Yes	63 (47.7)	174 (60.0)	1	
No	69 (52.3)	116 (40.0)	1.64 (1.09,2.49)	
Financial problem				
Yes	31 (23.5)	32 (11.0)	2.48 (1.44,4.27)	2.26 (1.26, 4.06)
No	101 (76.5)	258 (89.0)	1	1
Go to traditional healer				
Yes	17 (12.9)	17 (5.9)	2.37 (1.17,4.81)	
No	115 (87.1)	273 (94.1)	1	
Reason for first consultation/visit of health facilities/				
Severely ill with the diseases				
Yes	117 (88.6)	232 (80.0)	1.95 (1.06,3.59)	2.09 (1.10, 3.98)
No	15 (11.4)	58 (20.0)	1	1

## Discussion

In the current study, the median health care seeking delay was 30 days (IQR = 39). This is similar or shorter than several studies conducted in Africa, including Ethiopia [7, 8, 12, 19, 20], Zimbabwe [21], Uganda [22], Nigeria [23], Angola [24], Ghana [17], and Mozambique [16] which ranges from 1 to 3 months. Likewise, it is similar to the average health care seeking delay in LMICs which was 31.7 days [4]. However, the finding of the present study is much longer than the recommended 2 weeks [25]. In addition, it is still higher than in Malawi (14 days) [26], Cameroon (14 days) [20], and India (18.4 days) [27]. This implies that health care seeking delay is still very high and much is expected to be done to reduce health care seeking delay.

The results of this study revealed that occupation, knowledge, health facility visited first, seeking treatment from traditional or religious healers, reason for not seeking treatment early from health facility and reasons for consultation were significantly associated with health care seeking delay.

Regarding the occupational status; farmers were more likely to delay compared to their counterparts. This finding is in agreement with study findings done in Uganda, where farmers were experiencing unacceptable patient delay compared to the rest of the respondents [28]. Similarly farming has been reported as a risk factors for health care seeking delay in similar study in Mozambique [16]. This could be due to lack of awareness on tuberculosis among farmers which eventually affects health seeking behavior.

Findings of this study revealed that limited knowledge about PTB was significant risk factor for health care seeking delay. Similar findings have been documented in China, Ethiopia, Mozambique and Malawi [19, 26, 29–31], in which limited PTB knowledge has been reported as a risk factor for health care seeking delay. This could be due to the fact that limited knowledge about tuberculosis affects health seeking behavior which result in unacceptable health care seeking delay.

Health care seeking delay was associated with the type of health facility visited first; patients who consulted public health center were less likely to experience health care seeking delay compared to those who consulted to the public hospital. This finding is not in agreement with similar studies conducted elsewhere [15, 32]; in which patients who visited public health center were more likely to experience health care seeking delay. But a study by Sabawoon et al. revealed similar finding that patients diagnosed at health center were less likely to experience longer health care seeking delay [33]. This implies that strengthening health centers as tuberculosis diagnosis and treatment center could contribute a lot in reducing delay.

It was noticed that seeking treatment from traditional or religious healers before visiting the health facilities was another factor associated with health care seeking delay; seeking treatment from non-formal providers (traditional healers, religious healer/holy water) was a significant risk factor for longer health care seeking delay. This finding is in agreement with several studies conducted elsewhere [8, 9, 12, 14, 16, 33]. In the present study, financial problem was cited as a reason for not seeking health care from health facilities. This is in agreement with studies in Asia, Ghana, India, Ethiopia, and others [11, 17, 27, 32, 34].

According to the findings of this study, severity of the disease as a reason for consultation from a health facility was significantly associated with health care seeking delay. This might be due to the fact that severely ill patients immediately visit the health facility considering their illness were at it's terminal stage. This was also supported by study that severety of illness prevents PTB patients from visiting the health facility was considered as a reason for delay [12]. Besides to this, hemoptysis which is a proxy indicator of severety of tuberculosis was a risk factor for health care seeking delay in a systematic review and meta-analysis study in Asia [32]. This finding depicted that there was a delay in health care seeking behavior among tuberculosis patients.

## Conclusion

In conclusion, the study showed the magnitude of health care seeking behavior among pulmonary tuberculosis patients was much delayed. Occupation; being a farmer, limited knowledge, type of health facility consulted first; public health center, consulting traditional or religious healers first, reason for not seeking treatment early from health facilities; financial problem, and reason for first consultation; being severely ill with the diseases were independent predictors of health care seeking delay. Effective implementation of the strategy requires enhanced resource mobilization to health centers to equip with diagnostic medical modalities. Meanwhile the health workers should work rigorously on community awareness creation on the benefits of early health care seeking for any medical illness.

## Abbreviations

AFB: Acid-Fast Bacilli
AOR: Adjusted odds ratio
ART: Anti-retroviral treatment
CI: Confidence interval
COR: Crude odds ratio
DOT: Directly observed treatment
DOTS: Directly Observed Treatment Short Course
IQR: Inter-quartile range
LMICS: Low and middle income countries
MDRTB: Multi-Drug Resistant TB
PTB: Pulmonary tuberculosis
SD: Standard deviation
SNPTB: Smear negative pulmonary tuberculosis
SPPTB: Smear positive pulmonary tuberculosis
TB: Tuberculosis
WHO: World Health Organization
XDRTB: Extensively Drug Resistant TB
